# Ischemic Stroke as a Possible Initial Manifestation of Primary Sjögren's Syndrome: A Case Report

**DOI:** 10.7759/cureus.113812

**Published:** 2026-08-02

**Authors:** Bashayer I Alkhalifah, Dalya A Abualsaud

**Affiliations:** 1 Internal Medicine, Prince Saud Bin Jalawi Hospital, Al Mubarraz, SAU

**Keywords:** autoimmune vasculitis, case report, central nervous system involvement, ischemic stroke, primary sjögren's syndrome

## Abstract

Primary Sjögren's syndrome (pSS), a chronic autoimmune disorder primarily affecting the exocrine glands, typically presents with sicca symptoms. Neurological involvement in pSS is uncommon as an initial presentation. Ischemic stroke, in particular, is a rare manifestation, while seizures are more commonly secondary to underlying structural or inflammatory central nervous system involvement. Here, we report a 41-year-old female who presented with a generalized tonic-clonic seizure preceded by left-sided tinnitus and associated with tongue biting, ocular deviation, and postictal confusion. Notably, seven years earlier, she had experienced a neurological event characterized by sudden-onset left-sided weakness and mouth deviation, with neuroimaging at that time reportedly demonstrating an acute infarction in the right temporoparieto-occipital region. During the current admission, brain imaging demonstrated chronic encephalomalacia and gliosis in the same region without evidence of acute infarction. Electroencephalography revealed focal epileptiform discharges in the left temporal lobe. Extensive evaluation of stroke etiologies was unrevealing. Subsequent immunological testing revealed positive anti-Sjögren's syndrome-related antigen A (SSA)/Ro and antinuclear antibodies (ANA). Although sicca symptoms were not reported at the time of the initial neurological presentation, the patient subsequently developed dry eye and dry mouth symptoms, prompting further evaluation with Schirmer's test and labial salivary gland biopsy, which confirmed the diagnosis of pSS. This case highlights the importance of considering autoimmune disorders, including pSS, in selected patients with cryptogenic neurological manifestations. It also underscores the diagnostic challenges posed by atypical presentations in the absence of classic sicca symptoms. Further studies are needed to better understand the relationship between pSS and cerebrovascular disease.

## Introduction

Primary Sjögren's syndrome (pSS) is an autoimmune exocrinopathy characterized by inflammatory lymphocytic infiltration of the exocrine glands, predominantly salivary and lacrimal glands [1]. With potential multisystem involvement, extraglandular complications occur in 70% of patients and commonly involve the joints, lungs, skin, and peripheral nervous system (PNS). Rarely, central nervous system (CNS) involvement may also be present [2]. Neurological manifestations, including ischemic stroke, have been reported in pSS and may be related to mechanisms such as autoimmune-mediated vasculopathy, endothelial dysfunction, or chronic systemic inflammation. Here, we report a rare case of ischemic stroke as a possible initial presentation of pSS, with a suspected underlying autoimmune-mediated vasculopathy.

## Case presentation

A 41-year-old previously healthy female presented to the emergency department with a generalized tonic-clonic seizure. The episode began with left-sided tinnitus. The tinnitus was reported as left-sided; however, detailed characterization, including pitch and pulsatile nature, was not documented at the time of presentation. This was followed by loss of consciousness and generalized convulsions, characterized by tongue biting and upward ocular deviation, and was subsequently followed by postictal confusion. Notably, the patient had a prior neurological event seven years earlier, characterized by sudden-onset left-sided weakness and deviation of the mouth. Neuroimaging performed at that time reportedly confirmed an acute infarction in the right temporoparieto-occipital region, consistent with ischemic stroke. In contrast, current brain imaging demonstrates chronic encephalomalacia/gliosis in the same region, with no diffusion restriction or evidence of acute infarction.

During admission, the patient underwent a comprehensive diagnostic evaluation for acute ischemic stroke. Initial investigations, including transthoracic echocardiography, carotid Doppler ultrasonography, and autoimmune workup, including anti-neutrophil cytoplasmic antibody (ANCA) testing, were unremarkable. She was managed acutely with antiplatelet therapy, statins, and levetiracetam, and subsequently referred for further evaluation through a multidisciplinary approach involving neurology and rheumatology teams.

Neurological examination

On neurological examination, the patient was alert and oriented with no cranial nerve deficits, cerebellar signs, or sensory abnormalities. The motor strength was preserved bilaterally with a power of 5/5 in all extremities. No hemiparesis was observed. Although no recurrent clinical seizures were observed during hospitalization and there was no prior diagnosis of epilepsy, the electroencephalographic findings demonstrated focal epileptiform activity. In the context of prior cortical infarction and gliotic changes, the seizure was considered most consistent with a late post-stroke seizure.

Laboratory evaluation revealed the following: hemoglobin (Hgb) of 12.7 g/dL, mean corpuscular volume (MCV) of 87.8 fL, white blood cell (WBC) count of 4.6 × 10⁹/L, and platelet count of 179 × 10⁹/L. Coagulation studies were within normal limits: prothrombin time (PT) 10.6 seconds, partial thromboplastin time (PTT) 26.5 seconds, and international normalized ratio (INR) normal (Table 1).

**Table 1:** Initial hematological and coagulation evaluation

Test	Result	Reference Range
White Blood Cell Count (WBC)	4.6 × 10⁹/L	4-10 × 10⁹/L
Hemoglobin (Hgb)	12.7 g/dL	13-17 g/dL
Hematocrit (Hct)	0.39	0.40-0.50 (40-50)%
Mean Corpuscular Volume (MCV)	87.8 fL	80-95 fL
Mean Corpuscular Hemoglobin (MCH)	28.3 pg	27-32 pg
Platelet Count	179 × 10⁹/L	150-450 × 10⁹/L
International Normalized Ratio (INR)	0.76	0.8-1.1
Prothrombin Time (PT)	10.6 sec	9.5-15 sec
Partial Thromboplastin Time (PTT)	26.5 sec	26-40 sec

Neuroimaging and electrodiagnostic findings

A non-contrast CT head was performed early during the current admission as part of the initial evaluation. It demonstrated a well-defined hypodensity consistent with chronic encephalomalacia with associated volume loss in the affected region, correlating with the patient's prior infarction, with no features suggestive of acute ischemia (Figure 1).

**Figure 1:**
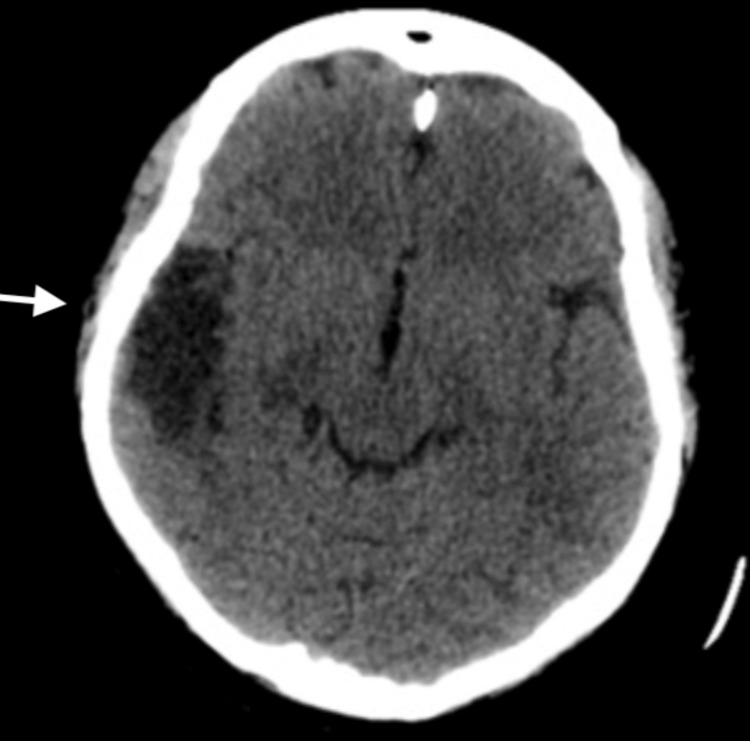
Non-contrast CT brain demonstrating right temporoparietal hypodensity (arrow), consistent with encephalomalacia and chronic ischemic insult

Subsequent brain magnetic resonance imaging (MRI) confirmed gliosis and volumetric loss of the right temporal lobe, extending to the periventricular region, consistent with an old ischemic event. Blooming artifacts were observed within the infarct core, likely reflecting chronic hemosiderin deposition. No diffusion restriction was observed, thereby ruling out acute infarction. The basal ganglia, thalami, brainstem, and cerebellum were within normal limits. There was no evidence of midline shift or hydrocephalus (Figure 2).

**Figure 2:**
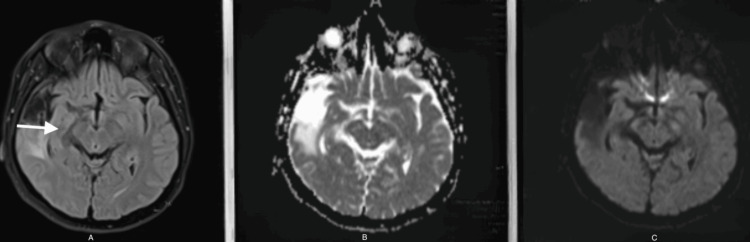
Brain MRI demonstrating right temporal lobe gliosis and volume loss, consistent with chronic ischemic insult (A) Fluid-attenuated inversion recovery (FLAIR) sequence showing hyperintense gliotic changes (arrow). (B) ADC map and (C) diffusion-weighted imaging (DWI) show no diffusion restriction.

Magnetic resonance angiography (MRA) of the brain revealed normal intracranial vasculature, including the anterior and middle cerebral arteries, vertebrobasilar system, and posterior circulation, with no evidence of stenosis, aneurysm, or arteriovenous malformations. Magnetic resonance venography (MRV) showed patent dural venous sinuses with preserved flow and no evidence of venous sinus thrombosis (Figure 3).

**Figure 3:**
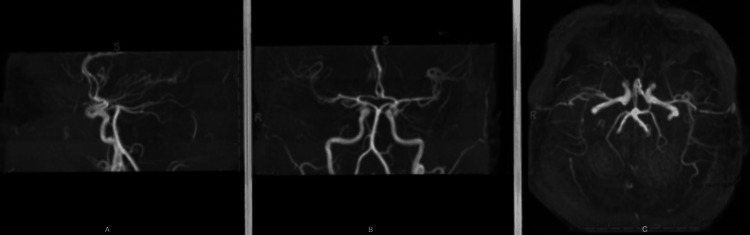
Magnetic resonance angiography (MRA) demonstrating normal intracranial arterial circulation without evidence of stenosis, aneurysm, or arteriovenous malformation (A, B) Magnetic resonance venography (MRV) (C) shows patent dural venous sinuses with preserved flow and no evidence of venous sinus thrombosis.

Carotid Doppler ultrasonography revealed normal flow velocity and no evidence of significant stenosis or atheromatous disease in the extracranial carotid arteries.

Transthoracic echocardiography demonstrated normal resting wall motion and preserved left ventricular systolic function with an estimated ejection fraction (EF) of 60%. Cardiac valves, including the mitral, aortic, and tricuspid valves, were structurally normal. Right-sided cardiac chambers (RA and RV) were normal in size and function. The estimated pulmonary artery pressure was 27 mmHg. The inferior vena cava measured 2 cm with normal respiratory collapse. The ascending aorta and descending aorta were unremarkable. No pericardial abnormality, intracardiac thrombus, or cardiac mass was identified.

Evaluation for patent foramen ovale was not performed during this admission. Electroencephalography (EEG) demonstrated focal epileptiform discharges localized in the left temporal lobe, consistent with a diagnosis of focal epilepsy. These findings indicate the presence of subclinical seizure activity independent of the chronic right-sided ischemic lesion identified on neuroimaging.

The current presentation was not considered a new ischemic stroke. Instead, it was interpreted as a late post-stroke seizure, likely secondary to cortical gliosis from the prior infarction.

Adult nerve conduction studies demonstrated normal sensory and motor nerve conduction across all the examined nerves. Specifically, the conduction velocities, amplitudes, and distal latencies of the bilateral median, ulnar, sural, peroneal, and tibial nerves were all within normal limits, while the F-wave latencies were also normal bilaterally. Overall, electrophysiological findings were normal, with no evidence of peripheral neuropathy.

Further laboratory workup revealed positive antinuclear antibodies (ANA) at 191.9 U/mL, and a markedly elevated anti-Sjögren's syndrome-related antigen A (SSA)/Ro antibody level at 118.48 U/mL, while anti-SSB (La) antibodies were within the normal limits at 9.63 U/mL. Rheumatoid factor (RF) (<20), anti-cyclic citrullinated peptide (anti-CCP) antibodies, and C- reactive protein (<20) were all negative. Complement levels were within normal limits, with C3 at 0.8 g/L (reference: 0.79-1.5) and C4 at 0.3 g/L (reference: 0.16-0.38). Lupus anticoagulant (LA) testing showed a clotting time of 34.5 seconds (reference: 33-47), which was within normal limits. Hepatitis B surface antigen (HBsAg), hepatitis C virus antibodies (HCV Ab), and HIV 1/2 Ag/Ab were negative. Antiphospholipid antibody screening was unremarkable, with negative results for anti-cardiolipin IgG, IgM, and anti-beta-2 glycoprotein I antibodies. ANCA testing was negative for antiproteinase 3 (PR3-ANCA) and antimyeloperoxidase (MPO-ANCA). Thrombophilia screening revealed normal protein S (75.6%), protein C (91.3%), and antithrombin III (84.9%) levels. Vitamin D deficiency was observed at 10.7 ng/mL. These results are presented in Table 2.

**Table 2:** Laboratory workup showing positive ANA and elevated anti-SSA (Ro) antibodies with otherwise unremarkable results ANA: antinuclear antibodies; anti-SSA: anti-Sjögren's syndrome-related antigen A; anti-SSB: anti-Sjögren's syndrome-related antigen B

Test	Result	Reference Range
Antinuclear Antibodies (ANA)	191.9 U/mL	—
Anti-SSA (Ro)	118.48 U/mL	—
Anti-SSB (La)	9.63 U/mL	—
Rheumatoid Factor (RF)	Negative (<20)	<20
Anti-cyclic Citrullinated Peptide (Anti-CCP)	Negative	Negative
C-reactive Protein (CRP)	Negative	Negative
Complement (C3)	0.8 g/L	0.79-1.5 g/L
Complement (C4)	0.3 g/L	0.16-0.38 g/L
Lupus Anticoagulant (LA)	34.5 sec	33-47 sec
Hepatitis B Surface Antigen (HBsAg)	Negative	Negative
Hepatitis C Virus Antibodies (HCV Ab)	Negative	Negative
HIV 1/2 Ag/Ab	Negative	Negative
Anti-cardiolipin IgG	Negative	Negative
Anti-cardiolipin IgM	Negative	Negative
Anti-β2 Glycoprotein I Ab	Negative	Negative
Anti-neutrophil Cytoplasmic Antibody, Antiproteinase 3 (PR3-ANCA)	Negative	Negative
Anti-neutrophil Cytoplasmic Antibody, Antimyeloperoxidase(MPO-ANCA)	Negative	Negative
Protein S	75.6% (Normal)	Normal
Protein C	91.3% (Normal)	Normal
Antithrombin III	95% (Normal)	Normal
Vitamin D	10.7 ng/mL	30-100 ng/mL

Despite the absence of any significant sicca symptoms at the time of the initial neurological presentation, the patient subsequently developed dry eye and dry mouth symptoms, prompting further evaluation using Schirmer's test and labial salivary gland biopsy. The biopsy revealed focal lymphocytic sialadenitis with a focus score ≥1, thereby fulfilling the American College of Rheumatology/European League Against Rheumatism (ACR/EULAR) classification criteria for pSS. After confirming the diagnosis, the patient was referred to the rheumatology team for further assessment by ophthalmology and oral medicine specialists. At the time of reporting, the patient had not received immunomodulatory therapy and remained under multidisciplinary follow-up.

## Discussion

Sjögren syndrome (SS) is a systemic autoimmune disease characterized by keratoconjunctivitis sicca (dry eyes) and xerostomia (dry mouth) [3]. This disease predominantly affects middle-aged women but can also occur in children, men, and the elderly [4,5].

The incidence rate of neurological complications in Sjögren syndrome ranges between 10% and 60%, and this complication is often due to peripheral neuropathy through small-vessel vasculitis [5]. Rarely, CNS involvement can occur, manifesting as psychiatric illnesses, cognitive deficits, meningoencephalitis, demyelinating disease, and transverse myelitis, amongst others [6]. Even more uncommonly, acute ischemic stroke, driven primarily by accelerated atherosclerosis and vasculitis, can develop in some cases [7].

In the present case, the patient experienced a cryptogenic ischemic stroke at a young age, followed by a generalized tonic-clonic seizure. Initial investigations failed to identify the conventional causes of stroke. However, subsequent autoimmune screening revealed high-titer ANA and anti-SSA/Ro antibodies. Although sicca symptoms were not reported at the time of the initial neurological presentation, the patient subsequently developed dry eye and dry mouth symptoms, prompting further evaluation. Histopathological examination of the salivary glands confirmed the diagnosis of pSS. The presence of focal epileptiform discharges on EEG and right temporoparietal gliotic changes on neuroimaging may reflect chronic CNS involvement and/or post-infarct neuronal remodeling.

Emerging evidence suggests that patients with pSS have an increased risk of both venous and arterial thromboembolic events. A large cohort study demonstrated significantly higher rates of cardiovascular disease, including myocardial infarction, venous thromboembolism, and cerebral infarction in patients with pSS compared to the general population, with the risk being particularly elevated in patients positive for both anti-SSA/Ro and anti-SSB/La antibodies [8]. Notably, among patients positive for anti-SSA/Ro and/or anti-SSB/La antibodies, the highest risk of cerebral infarction was observed after 10 or more years of disease duration. This increased risk may be related to chronic systemic inflammation, endothelial dysfunction, and type I interferon pathway activation associated with these autoantibodies [8].

Vitamin D deficiency was noted, with a serum level of 10.7 ng/mL. Although this finding was not considered directly related to the patient's neurological presentation, low vitamin D levels have been reported in autoimmune diseases, including primary Sjögren’s syndrome, and may contribute to immune dysregulation and increased disease activity.

Although traditional atherosclerotic risk factors were absent in our patient, the possibility of subclinical atherosclerosis as a contributing factor to the initial ischemic stroke was considered. Several studies have demonstrated increased subclinical atherosclerosis in patients with pSS, as evidenced by the elevated carotid intima-media thickness, pulse wave velocity, and reduced aortic distensibility [7]. These findings suggest a potential link between systemic inflammation and endothelial dysfunction in pSS. However, the clinical significance of these surrogate vascular markers remains unclear. Notably, a large-scale Taiwanese cohort study found no significant increase in the risk of atherosclerosis-related acute ischemic stroke among patients with pSS compared to the general population. This finding was supported by subsequent studies, casting doubt on the direct contribution of atherosclerosis to cerebrovascular events in pSS [9].

No radiological evidence of large-vessel involvement was identified, and additional investigations such as digital subtraction angiography and brain biopsy were not performed, as there were no clinical or radiological features strongly suggestive of active vasculitis. Therefore, a definitive diagnosis of CNS vasculitis could not be established. An autoimmune-mediated vasculopathy may represent a possible mechanism; however, this remains speculative and should be interpreted with caution.

The management of cerebral infarction secondary to vasculitis in pSS remains a clinical challenge owing to the rarity of CNS involvement and the limited availability of evidence-based guidelines. In cases in which autoimmune-mediated vascular inflammation is suspected, immunomodulatory therapy may be considered; however, current data supporting its efficacy have largely been derived from case reports and small observational studies. Therapeutic options include corticosteroids, steroid-sparing agents such as azathioprine or cyclophosphamide, and intravenous immunoglobulin (IVIG), although their role in treating the CNS manifestations of pSS is not well established. The use of biological agents, particularly rituximab, has shown variable outcomes, while current recommendations suggest reserving these therapies for patients with severe or refractory systemic diseases [10]. In our patient, although the diagnosis of pSS was confirmed in our patient, immunosuppressive therapy had not been initiated at the time of reporting. Given the presumed autoimmune etiology of initial ischemic stroke and the presence of subclinical epilepsy, a multidisciplinary approach is essential to guide long-term immunological and neurological management.

## Conclusions

This case highlights the importance of recognizing (pSS) as a potential associated condition in young adults presenting with cryptogenic ischemic stroke, particularly in the absence of conventional vascular risk factors. Such atypical presentations underscore the need for heightened clinical suspicion and comprehensive evaluation, including autoimmune screening, of cryptogenic stroke cases. Timely diagnosis is essential, as it allows for the early initiation of appropriate therapy, which may prevent recurrent neurological events and systemic complications.

The management of acute ischemic stroke related to pSS remains challenging owing to the limited clinical evidence and the rarity of such presentations. However, emerging data suggest a potentially beneficial role of immunomodulatory therapies and biological agents in controlling systemic inflammation and preventing further vascular events. Clinicians must be vigilant in identifying autoimmune causes of stroke, especially in younger patients, and adopt a multidisciplinary approach involving neurology, rheumatology, and internal medicine to ensure optimal patient care.
